# Lipid metabolism enzyme ACSVL3 supports glioblastoma stem cell maintenance and tumorigenicity

**DOI:** 10.1186/1471-2407-14-401

**Published:** 2014-06-04

**Authors:** Peng Sun, Shuli Xia, Bachchu Lal, Xiaohai Shi, Kil Sung Yang, Paul A Watkins, John Laterra

**Affiliations:** 1 MD Anderson Cancer Center, Houston, TX, USA; 2 Hugo W. Moser Research Institute at Kennedy Krieger, Baltimore, MD, USA; 3 Department of Neurology, Johns Hopkins School of Medicine, Baltimore, MD, USA; 4 Department of Oncology, Johns Hopkins School of Medicine, Baltimore, MD, USA; 5 Neuroscience, Johns Hopkins School of Medicine, Baltimore, MD, USA

**Keywords:** Lipid metabolism, ACSVL3, Glioblastoma, Cancer stem cell, Differentiation, Tumorigenicity

## Abstract

**Background:**

Targeting cell metabolism offers promising opportunities for the development of drugs to treat cancer. We previously found that the fatty acyl-CoA synthetase VL3 (ACSVL3) is elevated in malignant brain tumor tissues and involved in tumorigenesis. This study investigates the role of ACSVL3 in the maintenance of glioblastoma multiforme (GBM) stem cell self-renewal and the capacity of GBM stem cells to initiate tumor xenograft formation.

**Methods:**

We examined ACSVL3 expression during differentiation of several GBM stem cell enriched neurosphere cultures. To study the function of ACSVL3, we performed loss-of-function by using small interfering RNAs to target ACSVL3 and examined stem cell marker expression, neurosphere formation and tumor initiation properties.

**Results:**

ACSVL3 expression levels were substantially increased in GBM stem cell enriched neurosphere cultures and decreased after differentiation of the neurospheres. Down-regulating ACSVL3 with small inhibiting RNAs decreased the expression of markers and regulators associated with stem cell self-renewal, including CD133, ALDH, Musashi-1 and Sox-2. ACSVL3 knockdown in neurosphere cells led to increased expression of differentiation markers GFAP and Tuj1. Furthermore, ACSVL3 knockdown reduced anchorage-independent neurosphere cell growth, neurosphere-forming capacity as well as self-renewal of these GBM stem cell enriched neurosphere cultures. In vivo studies revealed that ACSVL3 loss-of-function substantially inhibited the ability of neurosphere cells to propagate orthotopic tumor xenografts. A link between ACSVL3 and cancer stem cell phenotype was further established by the findings that ACSVL3 expression was regulated by receptor tyrosine kinase pathways that support GBM stem cell self-renewal and tumor initiation, including EGFR and HGF/c-Met pathways.

**Conclusions:**

Our findings indicate that the lipid metabolism enzyme ACSVL3 is involved in GBM stem cell maintenance and the tumor-initiating capacity of GBM stem cell enriched-neurospheres in animals.

## Background

Targeting cancer specific metabolism represents an opportunity to develop novel, potentially selective and broadly applicable drugs to treat a multiplicity of cancer types. Malignant tissues require large amounts of lipid for membrane biosynthesis, energy, and signal transduction during tumor progression [1]. *De novo* fatty acid synthesis is the main means of fatty acid supply in cancers, therefore, enzymes involved in fatty acid metabolism have been implicated in cancer biology [2]. For example, overexpression of fatty acid synthase results in enhanced lipogenesis, a common feature in a variety of human cancers, including primary brain tumors [3,4]; and inhibiting fatty acid synthase or lipogenesis induces cancer cell death [5]. In addition to fatty acid synthase, several other enzymes involved in lipid metabolism have recently been shown to be involved in tumor growth and malignancy [6,7]. These data show that enzymes involved in lipid metabolism are potential therapeutic targets against cancers.

In the lipid metabolism cascade, addition of coenzyme A (CoA) to fatty acids is a fundamental initial step in the utilization of fatty acids for structural and storage lipid biosynthesis, signaling lipid protein acylation, and other metabolic processes [8]. Acyl-CoA synthetases (ACSs) are key enzymes for this fatty acid activation step [9]. ACS catalyzes an ATP-dependent multi-substrate reaction, resulting in the formation of fatty acyl-CoA. The overall reaction scheme is:

Fattyacid+ATP+CoA→Fattyacyl−CoA+PPi+AMP

Human cells contain 26 genes encoding ACSs [9,10]. Phylogenetically, ACSs are divided into at least four subfamilies that correlate with the chain length of their fatty acid substrates, although there is considerable overlap. There are short-chain ACS (ACSS), medium-chain ACS (ACSM), long-chain ACS (ACSL) and very long-chain ACS (ACSVL). Both ACSL and ACSVL isozymes are capable of activating fatty acids containing 16–18 carbons, which are among the most abundant in nature, but only the ACSVL family enzymes have significant ability to utilize substrates containing 22 or more carbons. Each ACS has a unique role in lipid metabolism based on tissue expression patterns, subcellular locations, and substrate preferences. For example, ACSL4 is overexpressed in breast, prostate, colon, and liver cancer specimens [11-13]. Among the multiple ACS members, two isozymes ACSL5 and ACSVL3, have been found important in gliomagenesis and malignancy [14,15].

Many solid malignancies, including glioblastoma multiforme (GBM), exhibit a cellular hierarchy containing subsets of tumor cells with stem-like features, which are currently believed to disproportionately contribute to tumor growth and recurrence [16,17]. These “cancer stem cells” display the capacity for long-term self-renewal, efficient propagation of tumor xenografts in experimental animals, the capacity for multi-lineage differentiation, and resistance to cytotoxic DNA-damaging agents [18,19]. Understanding the mechanisms that regulate cancer stem cell self-renewal and tumor-propagating potential could lead to new and more effective anti-cancer strategies.

The influence of lipid metabolism pathways on cancer stem cells has not been explored in great detail. ACSVL3 (alternatively designated as FATP3, SLC27A3) is one of the most recently characterized members of the ACS family [20]. Mouse ACSVL3 mRNA is found primarily in adrenal, testis, ovary, and developing brain; and ACSVL3 protein mainly localizes to subcellular vesicles that fractionate with mitochondria [20]. Compared with normal brain tissues, ACSVL3 expression levels are elevated in clinical GBM specimens and induced in GBM cells following the activation of oncogenic receptor tyrosine kinases. We previously reported that ACSVL3 supports tumor promoting capacity in human GBM [14], a biological property attributed to the cancer stem cell phenotype. This current study examines the expression and function of ACSVL3 in GBM stem cell enriched neurosphere isolates. We show that ACSVL3 functions to support GBM stem cell self-renewal and the capacity of GBM stem cells to propagate tumor xenografts. Our results suggest that targeting ACSVL3-dependent lipid metabolic pathways could be a strategy for inhibiting GBM stem cells and their capacity to support tumor growth and recurrence.

## Methods

### Reagents

All reagents were purchased from Sigma Chemical Co. (St. Louis, MO) unless otherwise stated. Hepatocyte growth factor (HGF) was a gift from Genentech (San Francisco, CA, USA). Epidermal growth factor (EGF) and basic fibroblast growth factor (bFGF) were purchased from Peprotech (Rocky Hill, NJ, USA). This study utilized discarded human pathological specimens from Johns Hopkins Neurological Operating Suite. Our use of de-identified pathological specimens as described here was reviewed by the John Hopkins IRB and designated to be “not human subjects research”.

### GBM neurosphere culture and differentiation

Human glioblastoma neurosphere lines HSR-GBM1A (20913) and HSR-GBM1B (10627) were originally derived by Vescovi and colleagues [16]. The GBM-DM14602 neurosphere line was derived from a glioblastoma at the University of Freiburg and kindly provided by Dr. Jaroslaw Maciaczy [21,22]. The primary neurospheres JHH612, JHH626 and JHH710 were derived from discarded glioblastoma surgical specimens at Johns Hopkins Hospital using the same methods and culture conditions as described in Galli et al. [16,23]. The primary neurosphere isolates were used at passage ≤ 10. All human materials were obtained and used in compliance with the Johns Hopkins IRB. GBM neurosphere cells were maintained in serum-free medium containing DMEM/F-12 (Life technologies, Carlsbad, CA), 1% BSA, EGF and FGF [16,24,25]. Cells were incubated in a humidified incubator containing 5% CO_2_ and 95% air at 37°C, and passaged every 4–5 days. Forced differentiation was performed according to the method of Galli et al. [16] with some modifications [26]. Briefly, the neurosphere cells were cultured on Matrigel (BD Biosciences, Bedford, MA, USA)-coated surfaces in medium containing bFGF (no EGF) for 2 days and then grown in medium containing 1% fetal bovine serum (FBS) without EGF/FGF for 3–5 days.

### Neurosphere transfection

Transient ACSVL3 knockdown was achieved using previously described ACSVL3 siRNA3 and ACSVL3 siRNA4 [20]. Targeted sequences of siRNA 3 and siRNA4 corresponded to the human ACSVL3 coding region (total 2430 bp) at bp1243-1263 and 1855–1875, respectively. Transfections of ACSVL3 siRNAs were performed with Oligofectamine (Life technologies) according to the manufacturer’s instructions. Fifteen nmol/L of siRNA was incubated with GBM neurosphere cells for 72 hours.

### Neurosphere-formation and clonogenic assays

Neurosphere cells were plated in six well plates. Cells were cultured in serum-free neurosphere medium for 5 days before being dissociated to single cell suspension and counted. For neurosphere formation assay, cells were grown for 5 days in medium containing EGF and FGF. Agarose (4%, Invitrogen) was then added to cultures to a final concentration of 1%. Immobilized neurospheres were stained with 1% Wright solution. For soft agar clonogenic assays, 1% agarose in DMEM was cast on the bottom of plastic six-well plates. Dissociated neurosphere cells (5 × 10^3^cells/well in 6 well plates) were suspended in neurosphere culture medium containing 0.5% agarose and placed on top of the bottom layer. Cells were incubated in neurosphere culture medium for 7–14 days and colonies were fixed and stained with 1% Wright solution. The number of spheres or colonies (>100 μm in diameter) was measured in three random microscopic fields per well by computer-assisted morphometry (MCID, Linton, Cambridge, England). For serial dilution of sphere-formation assay, cells were incubated with control or ACSVL3 siRNA3 for 48 h and plated at the density of 25, 50 and 100 cells/well in of 48 well/plates. Wells containing neurospheres diameter were counted after 3 days.

### Quantitative real time-PCR (qRT-PCR)

Total cellular RNA from GBM neurosphere cells was extracted using the RNeasy Mini kit (Qiagen, Germantown, MD, USA). The primer pairs used for amplifying genes of interest were: (1) ACSVL3: Forward primer 5′-cccagagtttctgtggctct-3′ and reverse primer 5′-ggacacttcagccagcaaat-3′ amplify a 256-bp intron-spanning ACSVL3 fragment; (2) nestin: forward primer 5′-aggatgtggaggtagtgaga-3′ and reverse primer 5′- ggagatctcagtggctctt-3′; (3) Musashi-1: forward primer 5′- gagactgacgcgccccagcc-3′ and reverse primer 5′-cgcctggtccatgaaagtgacg-3′; and (4) Sox-2: forward primer 5′- accggcggcaaccagaagaacag -3′ and reverse primer 5′- gcgccgcggccggtatttat -3′. Reverse transcription utilized MuLV Reverse Transcriptase and Oligo (dT) primers. Quantitative real-time PCR (qRT-PCR) was performed as we described in Ying et al. [21]. Relative expression of each gene was normalized to 18S RNA.

### Flow cytometry

The percentages of neurosphere cells expressing CD133 and ALDH were determined by analytical flow cytometry [21,26]. For the cell surface marker CD133, single-cell suspensions in 100 μl assay buffer (phosphate buffered saline pH 7.2, 0.5% bovine serum albumin, 2 mM EDTA) were incubated with 10 μl of phycoerythrin (PE)-conjugated anti-CD133 antibody (clone 293C3, Miltenyi Biotec, Auburn, CA) for 10 min in the dark at 4°C. Alternatively, single-cell suspensions were incubated ± diethylaminobenzaldehyde (DEAB) and then incubated in ALDH substrate (Stem Cell Technologies, Vancouver, Canada). The stained cells were analyzed on a FACScan (BD Biosciences). For sorting CD133+ from CD133− cells, neurosphere cells were incubated with microbead-conjugated CD133 antibodies and isolated with magnetic columns (Miltenyi Biotec).

### Immunoblotting and immunofluorescence staining

Immunoblotting analyses were performed as previously described [27]. The primary antibodies used were: anti-ACSVL3 (1:1000) [20]; anti-β-actin (1:6000); anti-GFAP (1:500, DAKO, Carpinteria, CA, USA) and anti-Tuj1 (1:1000, EMD).

For immunofluorescence staining, neurosphere cells were collected by cytospin onto glass slides, fixed with 4% paraformaldehyde for 30 min at 4°C, permeabilized with PBS containing 0.5% Triton X-100 for 5 min and stained with anti-GFAP and anti-Tuj1 antibodies according to the manufacturers’ protocols. Secondary antibodies were conjugated with Alexa 488 or Cy3 (Life Technologies). Coverslips were placed with Vectashield antifade solution containing 4′6-diamidino-2-phenylindole (Vector Laboratories, Burlingame, CA, USA). Immunofluorescent images were analyzed using Axiovision software (Carl Zeiss, Microscope, Thornwood, NY, USA).

### Intracranial xenograft mouse models

All animal protocols were approved by the Johns Hopkins Animal Care and Use Committee. Orthotopic tumor xenograft formation was assessed in 4- to 6-wk-old female mice as previously described [21]. HSR-GBM1A or HSR-GBM1B cells were transient transfected with ACSVL3 siRNAs for 3 days. Cell viability was determined by trypan blue dye exclusion. Equal numbers of viable cells (1×10^4^ cells/animal) in 5 μL PBS were injected unilaterally into the caudate/putamen of C.B-17 SCID/beige mice (n = 10) under stereotactic control [21]. The animals were sacrificed on post implantation week 10. Brains were removed, sectioned, and stained with H & E. Maximal tumor cross-sectional areas were measured by computer-assisted image analysis as previously described [28]. Tumor volumes were estimated according to the following formula: tumor volume = (square root of maximum cross-sectional area)^3^.

### Statistical analysis

Data were analyzed using Prism software (GraphPad, San Diego, CA, USA). When appropriate, two group comparisons were analyzed with a *t* test unless otherwise indicated. Multiple group comparisons were analyzed by one-way ANOVA with Bonferroni’s multiple comparison. All data are represented as mean value ± standard error of mean (SEM); *n* = 3 unless indicated otherwise. Significance was set at *P* < 0.05.

## Results

### ACSVL3 expression correlates inversely with differentiation of GBM stem cells

**Figure 1:**
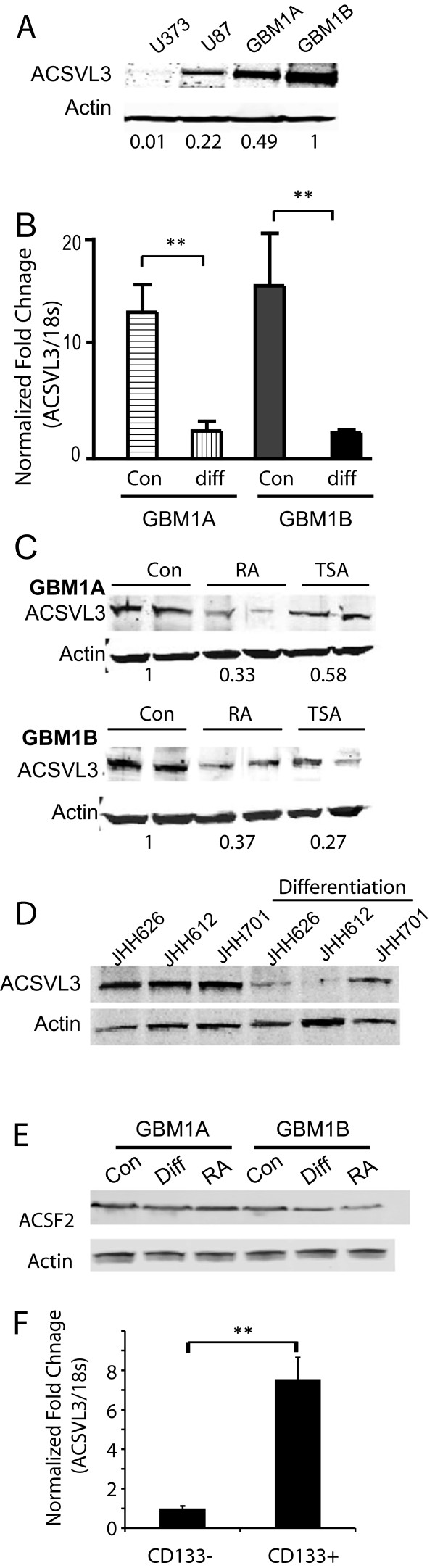
**ACSVL3 expression was decreased in differentiated GBM neurosphere cells. A**. Western blot analysis of ACSVL3 expression in adherent GBM cells maintained in serum (U373, U87) and in GBM neurosphere cells maintained in serum-free medium [HSR-GBM1A (GBM1A) and HSR-GBM1B (GBM1B)]. Blot was quantified by ImagJ and the fold change over actin is listed underneath. **B**. qRT-PCR analysis indicated that ACSVL3 mRNA level significantly decreased after GBM neurosphere cells were forced to differentiate (diff) by growth factor withdrawal and 1% serum. Total cellular RNA was extracted from cells 5 days under differentiation conditions. *Columns*, mean relative ratio of ACSVL3 to 18S RNA from triplicate determinations. **C**. GBM neurosphere cells (HSR-GBM1A and HSR-GBM1B) were cultured in neurosphere medium (Con) and treated with differentiating agents retinoic acid (RA, 10 μmol/L) or histone deacetylase inhibitor trichostatin A (TSA, 200 nmol/L) for 48 hours. Western blot analysis showed a ∼50-75% decrease in ACSVL3 protein following treatment with the two differentiating agents. Blots were quantified by ImageJ and the average fold changes over actin were listed underneath. **D**. Western blot analysis for ACSVL3 expression in low passage primary neurosphere cells (JHH626, JHH612 and JHH701) and their differentiated partners (diff) induced by growth factor withdrawal and 1% serum for 5 days. Differentiation resulted in decreased ACSVL3 expression in all three primary GBM neurosphere cultures. **E**. The expression of another member of the Acyl-CoA synthetase family, ACSF2, was not significantly altered in response to forced differentiation by serum- or RA. **F**. CD133+ and CD133− cells were isolated from GBM1A neurospheres using microbead-conjugated CD133 antibodies and magnetic columns (Miltenyi Biotec). Messenger RNAs were extracted from the two cell populations and subjected to qRT-PCR. Compared to CD133- cells, CD133+ cells expressed significantly higher levels of ACSVL3 (∼7.5 fold).*: *P* < 0.05; **: *P* < 0.01.

Human GBM neurosphere cultures that are enriched with cancer stem cells, including HSR-GBM1A, HSR-GBM1B, GBM-DM14602 and primary GBM neurosphere isolates from GBM patients, have been extensively characterized by us and others in terms of their stem cell marker expression, differentiation potential and tumor initiation capacity [16,21,24,25,29,30]. We compared ACSVL3 expression levels in both adherent GBM cell cultures maintained in serum-containing medium and in neurosphere cultures. Immunoblot analyses showed that ACSVL3 expression was found to be absent or lower in adherent GBM cell lines not enriched for GBM stem cells (i.e. U373 and U87, respectively,) in comparison to more elevated ACSVL3 expression in HSR-GBM1A and HSR-GBM1B neurosphere cells (Figure 1A). To determine if high ACSVL3 expression is associated with GBM stem cell properties, we examined ACSVL3 expression in GBM neurosphere cells following differentiating stimuli. ACSVL3 expression was diminished by ∼80% following forced differentiation (Figure 1B, *P* < 0.01). Treating GBM neurosphere cells with either of the differentiating agent all-trans retinoic acid (RA) or the histone deacetylace inhibitor trichostatin A (TSA) [21,25] also resulted in significant reductions (by 50-75%) in ACSVL3 protein levels (Figure 1C). Similar effects of forced differentiation on ACSVL3 expression levels were seen in multiple low passage primary GBM neurosphere isolates (Figure 1D). The effect of forced differentiation was specific for ACSVL3 since ACSF2, a related acyl-CoA synthetase family member that activates medium-chain fatty acids [20], was not affected by identical differentiation conditions (Figure 1E). The reduction in ACSVL3 expression with differentiation suggests that ACSVL3 preferentially associates with the stem-like cell subsets. Therefore, we used flow cytometer to separate and evaluate ACSVL3 expression in CD133+ and CD133- cells. Real-time PCR indicated that CD133+ cells expressed ∼7.5-fold higher ACSVL3 compared with CD133- cells (Figure 1F).

### ACSVL3 knockdown depletes GBM stem cell marker expression and promotes differentiation

**Figure 2:**
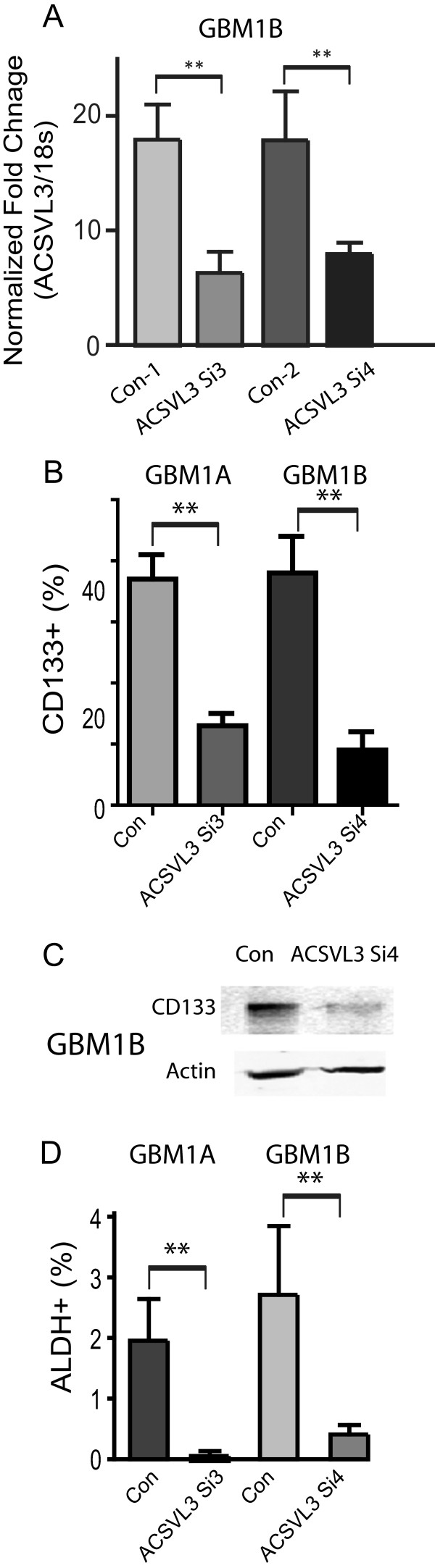
**ACSVL3 knockdown depleted GBM neurosphere cells expressing stem cell markers. A**. ACSVL3 expression was knocked down with two ACSVL3 siRNAs: ACSVL3 si3 and ACSVL3 si4. Quantitative RT-PCR (qRT-PCR) analysis indicated that compared to scrambled siRNA transfected cells (con), ACSVL3 mRNA level decreased ∼55-60% following ACSVL3 siRNA transfection for 72 hours in HSR-GBM1B cells. 18S was used as an internal control for qRT-PCR. *Columns*, mean relative ratio of ACSVL3 to 18S RNA from triplicate determinations. **B**-**D**. GBM neurosphere cells (HSR-GBM1A and HSR-GBM1B) were transiently transfected with ACSVL3 siRNAs and cultured in serum-free medium. Neurosphere cells were dissociated and subjected to flow cytometry to detect CD133-expressing cells **(B)** and ALDH-expressing cells **(D)**. Total cellular proteins from neurospheres were collected and subjected to immunoblotting analysis for CD133 expression **(C)**. ACSVL3 down-regulation significantly decreased the percentage of neurosphere cells with CD133+ and ALDH+. *: *P* < 0.05; **: *P* < 0.01.

To understand how ACSVL3 contributes to the phenotype of GBM neurosphere cells, we generated ACSVL3 knockdown GBM neurosphere cells by transiently transfecting the cells with two ACSVL3 siRNAs (si3 and si4) that target different regions of ACSVL3 mRNA. These siRNAs have previously been shown to inhibit ACSVL3 expression in adherent human GBM cells [14]. Quantitative RT-PCR (qRT-PCR) revealed that ACSVL3 si3 and ACSVL3 si4 inhibited ACSVL3 mRNA levels in GBM neurosphere cells by ∼60% and ∼55%, respectively (Figure 2A, *P* < 0.01).

**Figure 3:**
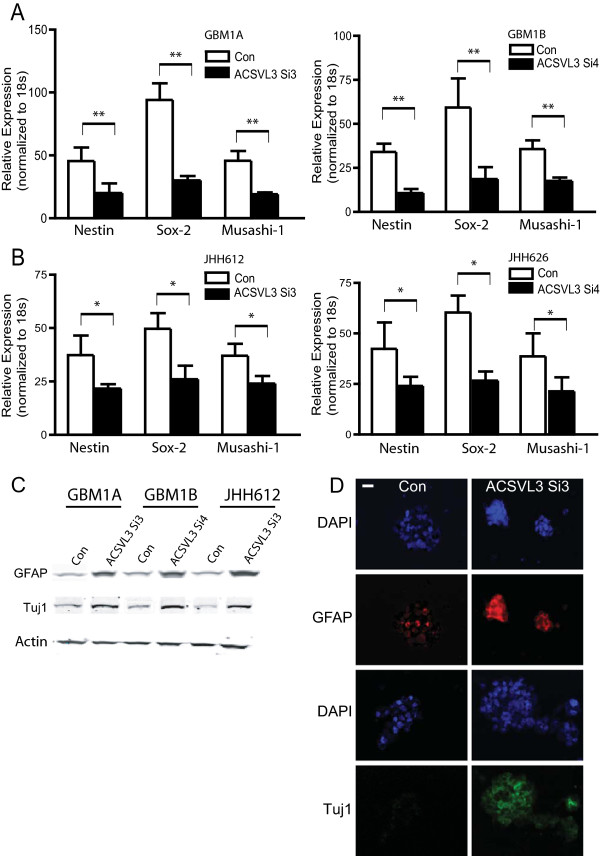
**ACSVL3 knockdown reduced stem cell marker expression and induced differentiation of GBM stem cell enriched neurospheres. A**-**B**. HSR-GBM1A, HSR-GBM1B cells and low passage primary neurosphere cells (JHH612 and JHH626) were incubated with ACSVL3 siRNAs for 72 hours. Total cellular RNAs were extracted and subject to qRT-PCR to detect expression of stem cell markers nestin, sox-2 and Musashi. 18S was used as an internal control for qRT-PCR. ACSVL3 knockdown significantly inhibited stem cell marker expression in GBM stem cell enriched neurospheres. **C**. ACSVL3 knockdown promoted differentiation of GBM neurosphere cells. GBM neurosphere cells (HSR-GBM1A, HSR-GBM1B) and low passage primary neurosphere cells (JHH612) were transfected with ACSVL3 siRNA for 3 days followed by immunoblotting analysis to detect differentiation markers GFAP (astroglial marker) and Tuj1 (neuronal marker). ACSVL3 knockdown induced a 3-4-fold increase in GFAP expression and a 1.5-2-fold increase in Tuj1 expression, respectively. **D**. Immunofluorescence staining confirmed the increase of GFAP and Tuj1 expression following ACSVL3 knockdown. Neurospheres cells were collected by cytospin and then stained with anti-GFAP (red) and anti-Tuj1 (green) antibodies. Nuclei were stained with DAPI (blue). ACSVL3 knockdown induced an increase in GFAP and Tuj1 expression.

We examined the effects of ACSVL3 knockdown on neurosphere cell expression of stem cell specific markers. In HSR-GBM1A and 1B cells, the fraction of CD133^+^ cells decreased from ∼ 38% in control- transfected cells to ∼ 16% in cells receiving ACSVL3 siRNAs (Figure 2B, *P* < 0.01). Immunoblot analysis further confirmed that CD133 expression decreased substantially following ACSVL3 knockdown (Figure 2C). We also measured the expression of another stem cell marker, aldehyde dehydrogenase (ALDH). Quantitative Aldefluor flow cytometry assay revealed that the fraction of ALDH^+^ cells decreased ∼ 10-fold from ∼ 3.8% in controls to 0.4% in response to ACSVL3 siRNAs (Figure 2D, *P* < 0.01). ACSVL3 knockdown also reduced the expression of other markers and regulators associated with stem cell self-renewal, including Nestin, Sox-2, and Musashi-1 as determined by qRT-PCR (Figure 3A, *P* < 0.01). Similar effects of ACSVL3 knockdown on stem cell marker expression were observed in several low passage primary GBM neurosphere cells directly derived from patient samples (Figure 3B, *P* < 0.05).Since ACSVL3 expression is reduced following the forced differentiation of GBM neurospheres, we asked if ACSVL3 knockdown is sufficient to promote differentiation of cancer stem cells by examining the expression of the astroglial and neuronal lineage-specific markers GFAP and β-tubulin III (Tuj1). Expression levels of both differentiation markers were substantially increased 96 hours after ACSVL3 siRNA transfection (Figure 3C). GFAP expression increased ∼3-4 fold in HSR-GBM1A, HSR-GBM1B and JHH626 cells following ACSVL3 knockdown; and Tuj1 expression was induced 1.5-2 fold in these three cell lines. Immunofluorescence staining confirmed that GFAP and Tuj1 expression was relatively low in control transfected cells and increased after ACSVL3 knockdown (Figure 3D). These data suggest that ACSVL3 has a role in supporting the pool of GBM stem cells as ACSVL3 knockdown decreases stem cell marker expression and promotes differentiation.

### ACSVL3 knockdown inhibits GBM neurosphere growth and abrogates tumor propagating capacity of GBM stem cell enriched neurospheres

**Figure 4:**
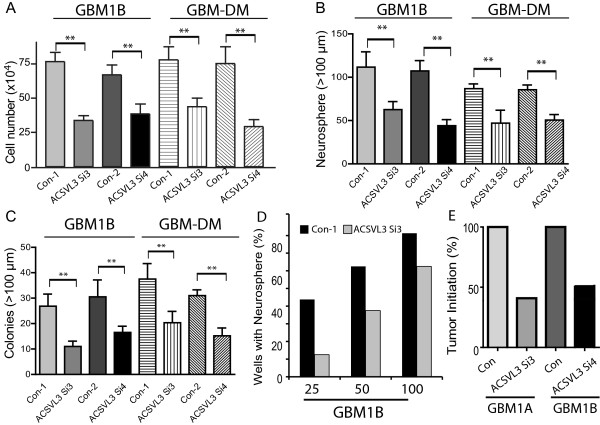
**ACSVL3 knockdown decreased GBM neurosphere cell growth and tumor initiation capacity of GBM neurosphere cells. A**. GBM neurosphere cells (HSR-GBM1B and GBM-DM14602) were transiently transfected with ACSVL3 siRNAs for 72 hours and cultured for 5–7 days. Neurosphere cell growth was determined by counting total cell number in cultures. Compared to control, there was a ∼ 45-55% and ∼37-45% cell number decrease in HSR-GBM1B and GBM-DM cells receiving ACSVL3 siRNAs, respectively. **B**. GBM neurosphere cells were transiently transfected with ACSVL3 siRNAs and cultured continuously for 14 days in neurosphere medium. Neurospheres were immobilized in agar and the number of neurospheres measuring bigger than 100 μm in diameter per low powered microscopic field was counted by computer-assisted morphometry MCID. ACSVL3 siRNA significantly inhibited neurosphere-forming ability of GBM neurosphere cells. **C**. Control or ACSVL3 knockdown GBM neurosphere cells were cultured in soft agar for 14 days before quantifying neurospheres number and size with MCID. ACSVL3 down-regulation significantly decreased clonogenicity of GBM neurosphere cells in soft agar. **D**. GBM1B neurosphere cells were incubated with scrambled siRNA and ACSVL3 siRNA3 for 48 h followed by serial dilution neurosphere assay. After counting live cells with trypan blue exclusion, single suspension neurosphere cells were plated at 25, 50 and 100 cells per plate into 48wells/plates. Wells containing neurospheres were counted after 3 days. **E**. ACSVL3 knockdown reduced tumor propagation of GBM stem cell enriched neurospheres. HSR-GBM1A or HSR-GBM1B cells were transfected with scrambled siRNA (con) or ACSVL3 siRNA for 3 days *in vitro*. Equal numbers of viable cells (1× 10^4^) were implanted into the caudate/putamen region of mouse brains (n = 10). After 10 weeks, the mice were sacrificed. Histological analysis (H & E staining) revealed that all the animals receiving control transfected cells developed intracranial tumors. In animals receiving ACSVL3 knockdown GBM neurosphere cells, only 40-50% of them developed detectable tumors.

To investigate the role of ACSVL3 in supporting GBM stem cell self-renewal, we examined GBM neurosphere cell growth and their sphere-formation capacity in response to ACSVL3 knockdown. Compared to control transfected cells, transient ACSVL3 knockdown significantly inhibited neurosphere cell growth by ∼45-55% in HSR-GBM1A and 1B cells (Figure 4A , *P* < 0.01). Neurosphere-forming capacity has been implicated as a biological marker of cancer stem cells since most cancer stem cells form large neurospheres in contrast to small neurospheres generated by progenitor cells. We therefore examined neurosphere size and number to determine the effects of ACSVL3 knockdown on cells displaying the stem-like phenotype. ACSVL3 knockdown reduced the number of neurospheres with a diameter >100 μm by ∼50% in both HSR-GBM1A and 1B cells (Figure 4B, *P* <0.01). ACSVL3 knockdown also significantly inhibited the formation of colonies in soft agar (clonogenicity, Figure 4C, *P* < 0.01). Similar results were found in GBM-DM14602 cells (Figure 4A-C). In addition, we performed serial dilution sphere-forming assays after ACSVL3 knockdown. ACSVL3 knockdown decreased the self-renewal capacity of GBM stem cells as evaluated by fewer neurospheres in limited dilution assays (Figure 4D).

A defining phenotype of cancer stem cells is their ability to propagate and maintain malignant tumors *in vivo*. We examined the effect of ACSVL3 knockdown on the orthotopic tumor propagating capacity of GBM neurosphere cells. HSR-GBM1A and GBM1B cells were treated with ACSVL3 siRNAs for 4 days in culture. Equal numbers of viable control and ACSVL3 siRNA-treated cells were implanted orthotopically into mice. ACSVL3 knockdown significantly reduced tumor initiation. All animals (n = 10) receiving control treated cells developed detectable intracranial tumors after 10 weeks. In contrast, only 40-50% of animals receiving ACSVL3 siRNA3-treated cells developed tumors (Figure 4E). This reduction in tumor initiation rate is consistent with the depletion of tumor-propagating cells in response to ACSVL3 knockdown.

### Induction of ACSVL3 expression by receptor tyrosine kinase (RTK) activation

**Figure 5:**
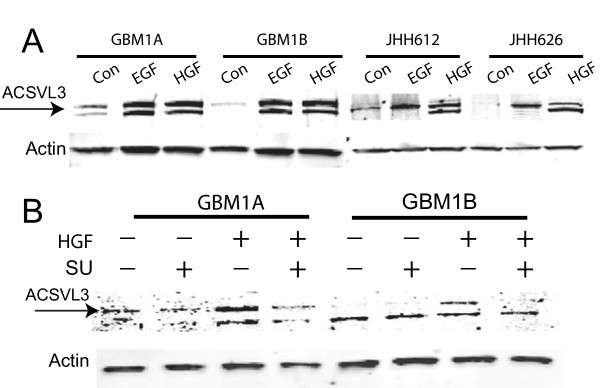
**Activation of receptor tyrosine kinase (RTK) signaling pathways induced ACSVL3 expression in GBM stem cell enriched neurospheres. A**. Incubation with EGF (30 ng/ml) or HGF (20 ng/ml) for 48 hours induced ACSVL3 expression in HSR-GBM1A, HSR-GBM1B and two primary low passage neurosphere cultures from GBM patients (JHH612, JHH626). **B**. Cells were pre-incubated with a small-molecule inhibitor of c-Met, SU11274 (20 μmol/L) for 6 hours and then treated with HGF (20 ng/mL) for 48 hour prior to immunoblotting analysis. Inhibition of the HGF/c-Met pathway reversed ACSVL3 induction by HGF.

We investigated the signaling pathways that mediate ACSVL3 expression in GBM stem cells. Our previous studies in U87 GBM cells indicate that RTK pathways such as HGF/c-Met and EGF/EGFR regulate ACSVL3 [14]. As the c-Met and EGFR pathways play an essential role in cancer stem cell maintenance [26], we asked whether the HGF/c-Met and EGF/EGFR pathways influence ACSVL3 expression in GBM stem cell enriched neurospheres. When the neurosphere cells were treated with EGF (50 ng/ml) or HGF (20 ng/mL) for 24 hours, an increase in ACSVL3 protein level was observed in HSR-GBM1A, GBM1B and in two primary low passage GBM neurosphere cultures, i.e. JHH612 and JHH626 (Figure 5A). Inhibition of the HGF/c-Met signaling pathway with a small molecule tyrosine kinase inhibitor SU11274 completely blocked HGF-mediated ACSVL3 up-regulation, confirming that multiple oncogenic RTK signaling pathways induce ACSVL3 expression in GBM neurosphere cells (Figure 5B).

## Discussion

A thorough understanding of cancer cell metabolism is critical to the identification of new targets for therapeutic intervention. Lipid metabolism in cancer is one area that has in general been under-studied. The identification of OA-519, a marker of poor prognosis in breast cancer, as fatty acid synthase two decades ago [31] sparked new interest in this area of cancer metabolism. Several new synthetic fatty acid synthase inhibitors have shown promise in preclinical studies [32,33]. However, to the best of our knowledge there are no current ongoing clinical trials testing drugs that target tumor lipid metabolism.

A significant issue in cancer therapeutics is that of recurrence and subsequent refractoriness to therapy. Tumor cells with stem-like features have been hypothesized to be, at least in part, responsible for these phenomena [16,17]. Thus, drugs that target stem-like cells would be an invaluable weapon in the treatment arsenal. Our previous work suggested that the acyl-CoA synthetase ACSVL3 was overproduced in human GBM and GBM cells in culture, and that decreasing the expression of this enzyme in GBM cells reduced both their malignant behavior in culture and their tumorigenicity in nude mice [14]. In this report, we show that expression of ACSVL3 is even more robust in cancer stem cell enriched neurospheres than in the cell population from which they were derived. Reducing ACSVL3 expression in these cells also decreased tumorigenicity in mice. Furthermore, differentiation of cancer stem cells with all-trans retinoic acid or Trichostatin A reduced ACSVL3 expression. Taken together, these observations indicate that ACSVL3 expression is associated with a highly undifferentiated phenotype and that therapeutic targeting this enzyme may be a promising anti-cancer therapy.

ACSVL3 is one of 26 acyl-CoA synthetases encoded by the human genome [34]. Acyl-CoA synthetases activate fatty acids to their coenzyme A thioesters, allowing subsequent entry into diverse metabolic pathways. RNA interference studies suggest that ACSVL3 is responsible for up to 30% of long-chain and very-long chain acyl-CoA synthetase activity in cells that endogenously express the enzyme [9]. Although this enzyme is also known as “fatty acid transport protein 3”, a role in fatty acid uptake could not be demonstrated experimentally [9]. Results presented here, and our previous work [14], show a correlation between ACLVL3 levels and cell growth rate, suggesting that this enzyme may provide fatty acid substrates required for bulk membrane phospholipid biosynthesis. Our current studies do not support this hypothesis (Shi and Watkins, unpublished); rather, a role in lipid signaling, possibly via phosphoinositide species and PI3 kinase signaling [14], seems more likely. The induction of ACSVL3 by RTK oncogenic pathways supports this notion, and indicates the importance of fatty acid metabolism in cancer stem cell maintenance. Activated fatty acid can regulate oncogenic signaling transduction pathways that are necessary for cell survival, proliferation, and differentiation [35], either directly or indirectly, by functioning as agonists of a number of G protein-coupled receptors, activating RTK downstream targets such as phosphatidylinositol 3-kinase/Akt and p44/42 mitogen-activated protein kinases, and stimulating phospholipase C/protein kinase. Elucidation of the specific downstream lipid metabolism pathways that are “fed” by ACSVL3 will provide new clues as to how this enzyme supports the malignant phenotype, and this is currently an area of active investigation in our laboratory.

Lipid metabolism has been linked to cellular differentiation mechanisms in some in vitro and in vivo models. ACSVL4 (or fatty acid transporter protein 4) has been shown to regulate keratinocyte differentiation [36]. Fatty acids and their metabolites can modulate stem cell self-renewal, survival, proliferation and differentiation by regulating gene expression, enzyme activity, and G protein-coupled receptor signal transduction [35]. Recent studies revealed that arachidonic acid (AA), eicosapentaenoic acid (EPA), and docosahexaenoic acid (DHA) may regulate the proliferation and differentiation of various types of stem cells. For example, both AA and EPA were the most potent inhibitors of proliferation of promyelocytic leukemic cells [37,38]. DHA or AA was found to promote the differentiation of neural stem cells into neurons by promoting cell cycle exit and suppressing cell death [39,40]. The role of fatty acid metabolism pathways in cancer stem cell differentiation has not been explored. To our knowledge, this is the first report showing that ACSVL3 regulates cancer stem cell phenotype and that ACSVL3 loss-of-function promotes cancer stem cell differentiation and inhibits tumor-initiation properties of cancer stem cells.

Our findings suggest that ACSVL3 is a potential therapeutic target worthy of further investigation. Findings reported here suggest that if identified, a small molecule inhibitor of ACSVL3 could inhibit the growth of GBM stem cells as well as non-stem tumor cells. Although there have been a few inhibitors of acyl-CoA synthetases reported [41-44], most are non-specific, and none that target ACSVL3 have been described. Research efforts to discover specific ACSVL3 inhibiters are also underway.

## Conclusions

Lipids regulate a broad spectrum of biological process that influences cell phenotype and oncogenesis. A better understanding of the biological function of lipid metabolism enzymes and cancer-specific lipid metabolic processes will enable us to identify new drug targets for cancer treatment. The results obtained in this study suggest that ACSVL3 is a potential therapeutic target in GBM. This is underlined by the fact that ACSVL3 is not essential for growth and survival of normal cells [20,45]. Developing pharmacological inhibitors of ACSVL3 will propel forward our effort to target lipid mechanism in brain tumors.

## Competing interests

The authors declare that they have no competing interests.

## Authors’ contributions

PS, SX: Conception and design, Collection and assembly of data, Data analysis and interpretation, Manuscript writing, Final approval; BL, XS, KY: Collection and assembly of data, Data analysis and interpretation, Final approval; PW, JL: Conception and design, Financial support, Administrative support, Data analysis and interpretation, Manuscript writing, Final approval.

## Pre-publication history

The pre-publication history for this paper can be accessed here:

http://www.biomedcentral.com/1471-2407/14/401/prepub

## References

[B1] Menendez JA Lupu R Fatty acid synthase and the lipogenic phenotype in cancer pathogenesis Nat Rev Cancer 2007 7 10 763 777 10.1038/nrc2222 17882277

[B2] Kuhajda FP Fatty-acid synthase and human cancer: new perspectives on its role in tumor biology Nutrition 2000 16 3 202 208 10.1016/S0899-9007(99)00266-X 10705076

[B3] Swinnen JV Brusselmans K Verhoeven G Increased lipogenesis in cancer cells: new players, novel targets Curr Opin Clin Nutr Metab Care 2006 9 4 358 365 10.1097/01.mco.0000232894.28674.30 16778563

[B4] Tong L Acetyl-coenzyme A carboxylase: crucial metabolic enzyme and attractive target for drug discovery Cell Mol Life Sci: CMLS 2005 62 16 1784 1803 10.1007/s00018-005-5121-4 PMC11139103 15968460

[B5] Lupu R Menendez JA Pharmacological inhibitors of Fatty Acid Synthase (FASN)–catalyzed endogenous fatty acid biogenesis: a new family of anti-cancer agents? Curr Pharm Biotechnol 2006 7 6 483 493 10.2174/138920106779116928 17168665

[B6] Brusselmans K De Schrijver E Verhoeven G Swinnen JV RNA interference-mediated silencing of the acetyl-CoA-carboxylase-alpha gene induces growth inhibition and apoptosis of prostate cancer cells Cancer Res 2005 65 15 6719 6725 10.1158/0008-5472.CAN-05-0571 16061653

[B7] Mashima T Seimiya H Tsuruo T De novo fatty-acid synthesis and related pathways as molecular targets for cancer therapy Br J Cancer 2009 100 9 1369 1372 10.1038/sj.bjc.6605007 19352381 PMC2694429

[B8] Watkins PA Fatty acid activation Prog Lipid Res 1997 36 1 55 83 10.1016/S0163-7827(97)00004-0 9373621

[B9] Watkins PA Very-long-chain acyl-CoA synthetases J Biol Chem 2008 283 4 1773 1777 10.1074/jbc.R700037200 18024425

[B10] Watkins PA Ellis JM Peroxisomal acyl-CoA synthetases Biochim Biophys Acta 2012 1822 9 1411 1420 10.1016/j.bbadis.2012.02.010 22366061 PMC3382043

[B11] Cao Y Dave KB Doan TP Prescott SM Fatty acid CoA ligase 4 is up-regulated in colon adenocarcinoma Cancer Res 2001 61 23 8429 8434 11731423

[B12] Monaco ME Creighton CJ Lee P Zou X Topham MK Stafforini DM Expression of long-chain fatty acyl-CoA synthetase 4 in breast and prostate cancers is associated with sex steroid hormone receptor negativity Transl Oncol 2010 3 2 91 98 10.1593/tlo.09202 20360933 PMC2847316

[B13] Sung YK Park MK Hong SH Hwang SY Kwack MH Kim JC Kim MK Regulation of cell growth by fatty acid-CoA ligase 4 in human hepatocellular carcinoma cells Exp Mol Med 2007 39 4 477 482 10.1038/emm.2007.52 17934335

[B14] Pei Z Sun P Huang P Lal B Laterra J Watkins PA Acyl-CoA synthetase VL3 knockdown inhibits human glioma cell proliferation and tumorigenicity Cancer Res 2009 69 24 9175 9182 10.1158/0008-5472.CAN-08-4689 19920185 PMC2795097

[B15] Yamashita Y Kumabe T Cho YY Watanabe M Kawagishi J Yoshimoto T Fujino T Kang MJ Yamamoto TT Fatty acid induced glioma cell growth is mediated by the acyl-CoA synthetase 5 gene located on chromosome 10q25.1-q25.2, a region frequently deleted in malignant gliomas Oncogene 2000 19 51 5919 5925 10.1038/sj.onc.1203981 11127823

[B16] Galli R Binda E Orfanelli U Cipelletti B Gritti A De Vitis S Fiocco R Foroni C Dimeco F Vescovi A Isolation and characterization of tumorigenic, stem-like neural precursors from human glioblastoma Cancer Res 2004 64 19 7011 7021 10.1158/0008-5472.CAN-04-1364 15466194

[B17] Singh SK Hawkins C Clarke ID Squire JA Bayani J Hide T Henkelman RM Cusimano MD Dirks PB Identification of human brain tumour initiating cells Nature 2004 432 7015 396 401 10.1038/nature03128 15549107

[B18] Bao S Wu Q McLendon RE Hao Y Shi Q Hjelmeland AB Dewhirst MW Bigner DD Rich JN Glioma stem cells promote radioresistance by preferential activation of the DNA damage response Nature 2006 444 7120 756 760 10.1038/nature05236 17051156

[B19] Dirks PB Brain tumor stem cells: the cancer stem cell hypothesis writ large Mol Oncol 2010 4 5 420 430 10.1016/j.molonc.2010.08.001 20801091 PMC5527933

[B20] Pei Z Fraisl P Berger J Jia Z Forss-Petter S Watkins PA Mouse very long-chain Acyl-CoA synthetase 3/fatty acid transport protein 3 catalyzes fatty acid activation but not fatty acid transport in MA-10 cells J Biol Chem 2004 279 52 54454 54462 10.1074/jbc.M410091200 15469937

[B21] Ying M Wang S Sang Y Sun P Lal B Goodwin CR Guerrero-Cazares H Quinones-Hinojosa A Laterra J Xia S Regulation of glioblastoma stem cells by retinoic acid: role for Notch pathway inhibition Oncogene 2011 30 31 3454 3467 10.1038/onc.2011.58 21383690 PMC3955956

[B22] Wang SD Rath P Lal B Richard JP Li Y Goodwin CR Laterra J Xia S EphB2 receptor controls proliferation/migration dichotomy of glioblastoma by interacting with focal adhesion kinase Oncogene 2012 31 50 5132 5243 10.1038/onc.2012.16 22310282 PMC3349801

[B23] Chaichana K Zamora-Berridi G Camara-Quintana J Quinones-Hinojosa A Neurosphere assays: growth factors and hormone differences in tumor and nontumor studies Stem Cells 2006 24 12 2851 2857 10.1634/stemcells.2006-0399 16945995

[B24] Bar EE Chaudhry A Lin A Fan X Schreck K Matsui W Piccirillo S Vescovi AL DiMeco F Olivi A Eberhart CG Cyclopamine-mediated hedgehog pathway inhibition depletes stem-like cancer cells in glioblastoma Stem Cells 2007 25 10 2524 2533 10.1634/stemcells.2007-0166 17628016 PMC2610257

[B25] Sun P Xia S Lal B Eberhart CG Quinones-Hinojosa A Maciaczyk J Matsui W Dimeco F Piccirillo SM Vescovi AL Laterra J DNER, an epigenetically modulated gene, regulates glioblastoma-derived neurosphere cell differentiation and tumor propagation Stem Cells 2009 27 7 1473 1486 10.1002/stem.89 19544453 PMC2935595

[B26] Li Y Li A Glas M Lal B Ying M Sang Y Xia S Trageser D Guerrero-Cazares H Eberhart CG Quinones-Hinojosa A Scheffler B Laterra J c-Met signaling induces a reprogramming network and supports the glioblastoma stem-like phenotype Proc Natl Acad Sci U S A 2011 108 24 9951 9956 10.1073/pnas.1016912108 21628563 PMC3116406

[B27] Reznik TE Sang Y Ma Y Abounader R Rosen EM Xia S Laterra J Transcription-dependent epidermal growth factor receptor activation by hepatocyte growth factor Mol Cancer Res 2008 6 1 139 150 10.1158/1541-7786.MCR-07-0236 18234969 PMC2839502

[B28] Lal B Xia S Abounader R Laterra J Targeting the c-Met pathway potentiates glioblastoma responses to gamma-radiation Clin Cancer Res 2005 11 12 4479 4486 10.1158/1078-0432.CCR-05-0166 15958633

[B29] Wang SD Bar EE Chaudhry A Lin A Fan X Schreck K Matsui W Piccirillo S Vescovi AL DiMeco F Olivi A Eberhart CG EphB2 receptor controls proliferation/migration dichotomy of glioblastoma by interacting with focal adhesion kinase Oncogene 2012 31 50 5132 5143 10.1038/onc.2012.16 22310282 PMC3349801

[B30] Ying M Sang Y Li Y Guerrero-Cazares H Quinones-Hinojosa A Vescovi AL Eberhart CG Xia S Laterra J Kruppel-like family of transcription factor 9, a differentiation-associated transcription factor, suppresses Notch1 signaling and inhibits glioblastoma-initiating stem cells Stem Cells 2011 29 1 20 31 10.1002/stem.561 21280156 PMC3516843

[B31] Kuhajda FP Jenner K Wood FD Hennigar RA Jacobs LB Dick JD Pasternack GR Fatty acid synthesis: a potential selective target for antineoplastic therapy Proc Natl Acad Sci U S A 1994 91 14 6379 6383 10.1073/pnas.91.14.6379 8022791 PMC44205

[B32] Orita H Coulter J Lemmon C Tully E Vadlamudi A Medghalchi SM Kuhajda FP Gabrielson E Selective inhibition of fatty acid synthase for lung cancer treatment Clin Cancer Res 2007 13 23 7139 7145 18056164 10.1158/1078-0432.CCR-07-1186

[B33] Vazquez-Martin A Colomer R Brunet J Menendez JA Pharmacological blockade of fatty acid synthase (FASN) reverses acquired autoresistance to trastuzumab (Herceptin by transcriptionally inhibiting ‘HER2 super-expression’ occurring in high-dose trastuzumab-conditioned SKBR3/Tzb100 breast cancer cells Int J Oncol 2007 31 4 769 776 17786307

[B34] Watkins PA Maiguel D Jia Z Pevsner J Evidence for 26 distinct acyl-coenzyme A synthetase genes in the human genome J Lipid Res 2007 48 12 2736 2750 10.1194/jlr.M700378-JLR200 17762044

[B35] Das UN Essential fatty acids and their metabolites as modulators of stem cell biology with reference to inflammation, cancer, and metastasis Cancer Metastasis Rev 2011 30 3–4 311 324 22005953 10.1007/s10555-011-9316-x

[B36] Herrmann T van der Hoeven F Grone HJ Stewart AF Langbein L Kaiser I Liebisch G Gosch I Buchkremer F Drobnik W Schmitz G Stremmel W Mice with targeted disruption of the fatty acid transport protein 4 (Fatp 4, Slc27a4) gene show features of lethal restrictive dermopathy J Cell Biol 2003 161 6 1105 1115 10.1083/jcb.200207080 12821645 PMC2173002

[B37] Das UN Begin ME Ells G Fatty acid changes during the induction of differentiation of human promyelocytic leukemia (HL-60) cells by phorbolmyristate acetate Prostaglandins Leukot Essent Fatty Acids 1992 46 3 235 239 10.1016/0952-3278(92)90077-V 1508958

[B38] Finstad HS Kolset SO Holme JA Wiger R Farrants AK Blomhoff R Drevon CA Effect of n-3 and n-6 fatty acids on proliferation and differentiation of promyelocytic leukemic HL-60 cells Blood 1994 84 11 3799 3809 7949136

[B39] Kawakita E Hashimoto M Shido O Docosahexaenoic acid promotes neurogenesis in vitro and in vivo Neuroscience 2006 139 3 991 997 10.1016/j.neuroscience.2006.01.021 16527422

[B40] Varney ME Hardman WE Sollars VE Omega 3 fatty acids reduce myeloid progenitor cell frequency in the bone marrow of mice and promote progenitor cell differentiation Lipids Health Dis 2009 8 9 10.1186/1476-511X-8-9 19296839 PMC2669087

[B41] Hall AM Wiczer BM Herrmann T Stremmel W Bernlohr DA Enzymatic properties of purified murine fatty acid transport protein 4 and analysis of acyl-CoA synthetase activities in tissues from FATP4 null mice J Biol Chem 2005 280 12 11948 11954 10.1074/jbc.M412629200 15653672

[B42] Kim JH Lewin TM Coleman RA Expression and characterization of recombinant rat Acyl-CoA synthetases 1, 4, and 5. Selective inhibition by triacsin C and thiazolidinediones J Biol Chem 2001 276 27 24667 24673 10.1074/jbc.M010793200 11319222

[B43] Li H Black PN Chokshi A Sandoval-Alvarez A Vatsyayan R Sealls W DiRusso CC High-throughput screening for fatty acid uptake inhibitors in humanized yeast identifies atypical antipsychotic drugs that cause dyslipidemias J Lipid Res 2008 49 1 230 244 10.1194/jlr.D700015-JLR200 17928635

[B44] Van Horn CG Caviglia JM Li LO Wang S Granger DA Coleman RA Characterization of recombinant long-chain rat acyl-CoA synthetase isoforms 3 and 6: identification of a novel variant of isoform 6 Biochemistry 2005 44 5 1635 1642 10.1021/bi047721l 15683247

[B45] Stahl A Gimeno RE Tartaglia LA Lodish HF Fatty acid transport proteins: a current view of a growing family Trends Endocrinol Metab 2001 12 6 266 273 10.1016/S1043-2760(01)00427-1 11445444

